# Sensory Profiles Predict Symptoms of Central Sensitization in Low Back Pain: A Predictive Model Research Study

**DOI:** 10.3390/jcm13164677

**Published:** 2024-08-09

**Authors:** Pieter J. Gräper, Aldo Scafoglieri, Jacqueline R. Clark, Joannes M. Hallegraeff

**Affiliations:** 1 Experimental Anatomy Research Group, Department of Physiotherapy, Human Physiology and Anatomy, Faculty of Physical Education and Physiotherapy, Vrije Universiteit Brussel, Laarbeeklaan 103, 1090 Brussels, Belgium; aldo.scafoglieri@vub.be (A.S.); joannes.marinus.hallegraeff@vub.be (J.M.H.); 2 Department of Master Education, SOMT University of Physiotherapy, Softwareweg 5, 3821 BN Amersfoort, The Netherlands; 3 Pains & Brains, 8 Beach Grove, Omokoroa 3114, New Zealand; jacqueline.clark@vub.be; 4 Pain in Motion Research Group, Department of Physiotherapy, Human Physiology and Anatomy, Faculty of Physical Education and Physiotherapy, Vrije Universiteit Brussel, Laarbeeklaan 103, 1090 Brussels, Belgium

**Keywords:** back pain, Adolescent/Adult Sensory Profile, sensory processing, central nervous system sensitization, sensory thresholds

## Abstract

**Background**: Acute low back pain has a high prevalence, and when persisting into chronicity, it results in enormous socio-economic consequences. Sensory preferences may be key factors in predicting central sensitization as the main mechanism of nociplastic pain and chronicity. **Objectives**: Build a model to predict central sensitization symptoms using sensory profiles based on the PROGRESS framework. **Methods**: A Prognostic Model Research study was carried out to predict central sensitization symptoms at 12 weeks, using baseline sensory profiles, based on 114 patients with acute low back pain. Independent variables were sensory profiles, state and trait anxiety, age, duration, pain severity, depressive symptoms, and pain catastrophizing. **Results**: This model, based on continuous data, significantly predicts central sensitization symptoms at 12 weeks. It contains two significantly contributing variables: sensory profile Sensory Sensitive (unstandardized B-value = 0.42; *p* = 0.01) and trait anxiety (unstandardized B-value = 0.53; *p* ≤ 0.001). The model has a predictive value of R^2^ = 0.38. **Conclusions**: This model significantly predicts central sensitization symptoms based on sensory profile Sensory Sensitive and trait anxiety. This model may be a useful tool to intervene in a bottom–up and top–down approaches to prevent chronicity in clinical practice, including individual sensory preferences and behavioral responses to sensory stimulation in rehabilitation strategies.

## 1. Introduction

Low back pain (LBP) is the leading cause of disability worldwide, and, in contrast with a low disease burden, it has enormous socio-economic consequences [1]. According to prevalence projections for LBP, an increase of 36.4% is suggested for the coming 30 years [1]. In acute low back pain (ALBP), plastic alterations of the nervous system are triggered to decrease sensory thresholds because of threatening or actual tissue damage and painful experiences, which act as stressors on the nervous system [2,3]. After the initial decrease in sensory thresholds in the acute phase, lower thresholds and synaptic hyperexcitability return to previous levels [4,5]. However, when painful experiences persist beyond normal tissue healing time, nociceptive hyperexcitability becomes maladaptive and is described as central sensitization (CS) [3,6]. Persisting CS in the acute phase into the chronic phase acts as a contributor to the development of chronic pain conditions, such as chronic low back pain (CLBP), when ALBP persists over 12 weeks [6,7]. CS includes several related mechanisms of the peripheral and central nervous system adaptations, responsible for nociceptive faciliatory pathways and decreased function of endogenous analgesia, including sensory hypersensitivity, and, as a result, contributing to the development of chronic pain conditions [3].

**Figure 1:**
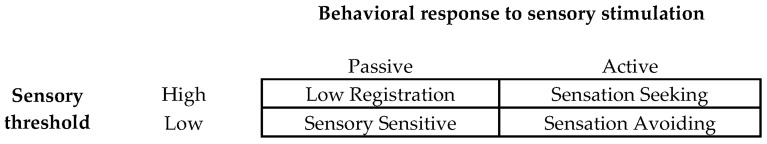
The sensory quadrants relating sensory threshold and behavioral responses to sensory stimulation, adapted from Dunn (1997) [8].

Trait sensory sensitivity levels are described by Dunn’s four-quadrant model of sensory processing that identifies sensitivity levels and behavioral responses to sensory stimulation based on individual sensory preferences [8,9,10,11] (Figure 1). In patients with CS, neurological sensory thresholds are lowered, and in combination with cognitive–emotional factors acting as psychological stressors, active (seeking or avoiding) or passive (non-responsive) behavioral responses are reinforced [3,10,12,13]. When lower neurological sensory thresholds are present, overstimulation can easily occur, and individual preferences for sensory stimulation are violated [8,12]. This can be reinforced when behavioral responses to sensory stimulation do not cohere with individual sensory preferences on top of the lower sensory thresholds [10]. As a result, a passive response, actively seeking or avoiding sensory stimulation, can become maladaptive in patients with ALBP and contribute to the development of CLBP as it facilitates CS [3,12].

Sensory profiles are a research-based framework to assess sensory processing of taste/smell, movement, vision, touch, activity levels, and auditive input and evaluate behavioral responses to sensory experiences of everyday life [8,9]. It can be hypothesized that maladaptive behavior, based on individual sensory profiles, which assess sensory thresholds and predict behavioral responses to sensory stimulation, contributes to the development of CS as the main mechanism behind nociplastic pain in chronic pain conditions and predicts poor outcomes [7,10,13]. Sensory profiles are established prognostic factors in the persistence of CS symptoms in the LBP population; prognostic model research may validate and evaluate the predictive role of sensory profiles over time [13,14]. Therefore, this study aims to evaluate the prognostic value of sensory profiles, adjusted with known factors in patients with acute low back pain, to assess the risk of developing CS symptoms at 12 weeks.

## 2. Materials and Methods

### 2.1. Study Design

The design of this study is a type III prognostic model research by the PROGRESS framework. The design was a multi-centered prospective cohort study, building a model to predict CS symptoms at 12 weeks by baseline sensory profiles. The four sensory profiles were adjusted by established prognostic factors. The Strengthening the Reporting of Observational Studies in Epidemiology (STROBE) Statement (STROBE) was used as a guideline, and the Transparent Reporting of a multivariable prediction model for Individual Prognosis or Diagnosis (TRIPOD) statement and the Reporting Recommendations for Tumor Marker Prognostic Studies (REMARK) checklist were used in reporting this study [14,15,16,17].

### 2.2. Ethics

The Medical Ethics Committee of the University Hospital Brussels, Belgium, gave their ethical approval to this project on 13 April 2022, trial number B.U.N.: 1432021000708. This study is registered at clinicaltrails.gov and can be identified by ClinicalTrials.gov Identifier: NCT05097235, following the declaration of Helsinki (revision 2013) [18].

### 2.3. Setting and Participants

Participants were consecutively recruited from two primary care physiotherapy practices in the Netherlands; one clinical practice is situated in a city, and the other is in a village. The inclusion criteria were the patients with ALBP (<6 weeks) that could not be attributed to an underlying structural lesion or specific disease, without structural or specific cause explaining the pain, with or without radiating pain, aged 18 to 60 years, and with a pain-free episode for at least 12 weeks before the onset of their current back pain episode [19]. They were also required to be able to read and understand the Dutch language. Exclusion criteria were a structural spinal problem or specific cause of LBP and when other parts of the body radiated the pain [19].

### 2.4. Data Collection and Measurement Procedures

Data collection was carried out from May 2022 to June 2023. Patients were consecutively included, and a researcher evaluated eligibility. Before participation, the study protocol was expressed verbally and in writing, and subsequently, informed consent was obtained. To avoid bias, baseline measurements were obtained before history taking and physical examination. In all participants, the sequence of measurements was identical, and ambiguity was clarified with minimal information. To protect the data, pseudo-anonymization was implemented by replacing direct participant identifiers with a pseudonym, initials of the physiotherapist who was obtaining the data, and inclusion number by the specific physiotherapist, resulting in the following form: XX/00. After obtaining the data from baseline and follow-up measures, the participant identifier (XX/00) changed into consecutive numbers to further improve pseudo-anonymization. Individual participants can only be identified by the primary investigator according to the Vrije Universiteit Brussel procedures. Confidentiality and anonymity were guaranteed by storing all records in a secure area, and an encrypted electronic database was used for all encoded data. Baseline measurements were obtained from the Adolescent/Adult Sensory Profile (AASP), the Numerical Pain Rating Scale (NPRS), the State-Trait Anxiety Inventory (STAI), the Beck Depression Inventory (BDI), the Pain Catastrophizing Scale (PCS), and after 12 weeks, the Central Sensitization Inventory (CSI). Usual care physiotherapy was applied to all participants [20]. The first measurement (t0/baseline) was within 6 weeks after onset of LBP, and the outcome measurement was 12 weeks after (t1/follow-up).

### 2.5. Measurements

#### 2.5.1. Adolescent/Adult Sensory Profile (AASP)

The AASP, as the main outcome variable, identifies 4 sensory profiles and their sensory thresholds as taste, smell, sight, hearing, touch, activity stimulation, and the individual’s adaptive responses (passive or active) to being over- or under-stimulated by sensory information [8,21]. The sensory profiles can be visualized as 4 quadrants, according to Dunn (1997), based on the level of sensory threshold and passive or active behavioral response to sensory stimulation [8]. The 4 sensory profiles include Low Registration with a high threshold and a passive response; Sensation Seeking, with a high sensory threshold actively seeking stimulation; Sensory Sensitive, with a low sensory threshold and a passive response; and Sensation Avoiding, with a low sensory threshold actively avoiding stimulation [8]. Each sensory profile score ranges from 15 to 75 [8,9,22]. Sensory profile scores are translated into 1 out of 5 categories (much lower than most people; lower than most people; like most people; higher than most people; much higher than most people) based on normative values [8,9,22]. Sensory hypo- of hypersensitivity traits are indicated if a sensory profile score is ±1 of standard deviation (SD), beyond the normative values [9], with an internal consistency of Cronbach’s alpha 0.91–0.92, and test–retest reliability ICC = 0.82–0.87 (95%CI = 0.74–0.91), [11]. The AASP is a reliable and valid measure to assess sensory profiles in the LBP population [11]. The sensory profiles within the AASP demonstrate considerable agreement, as construct validity correlates positively and significantly for Low Registration Pearson’s r = 0.33–0.45; Sensory Sensitive Pearson’s r = 0.29–0.57, and Sensation Avoiding Pearson’s r = 0.29–0.50, with the constructs of depression, anxiety (trait and state), helplessness, catastrophizing, rumination, and disability, but Sensation Seeking exhibited a negative correlation of Pearson’s r = −0.017; −0.06 [11].

#### 2.5.2. Known Factors

Known prognostic factors for LBP were used as adjusting variables [23]. They consist of age, duration of LBP, and the following Patient Reported Outcome Measures:

##### Numeric Pain Rating Scale (NPRS)

The Numeric Pain Rating Scale consists of an 11-point scale, ranging from 0 (no pain at all) to 10 (most extreme pain imaginable) to measure pain experiences, with a minimal important change of 2 points. The accuracy is fair to excellent, with an Area Under the Curve ranging from 0.72 to 0.92 [24].

##### State-Trait Anxiety Inventory (STAI-DY1, STAI-DY2)

The authorized Dutch translation of the State Anxiety Inventory (STAI-DY1) assesses anxiety as a momentary emotion to a threatening stimulus, and the Trait Anxiety Inventory (STAI-DY2) assesses anxiety as a consistent reaction being stable throughout life [23]. STAI-DY1 and STAI-DY2 consist of 20 statements each, scoring on a 4-point scale (ranging 20–80), and can be classified as “no or low anxiety” scoring 20–37, “moderate anxiety” scoring 38–44, and “high anxiety” scoring 45–80, with well-reported reliability and validity (Cronbach’s alpha = 0.90) [25].

##### Becks Depression Inventory (BDI)

The BDI is a commonly used 21-item self-reported questionnaire (ranging 0–63) and assesses depressive symptoms by the DSM-IV criteria for depressive disorders containing items on affective, cognitive, and somatic subscales [26,27,28,29,30,31].

##### Pain Catastrophizing Scale (PCS)

The PCS measures pain-relevant helplessness, rumination, magnifying, and catastrophizing in clinical and non-clinical populations [32,33].

#### 2.5.3. Central Sensitization Inventory (CSI)

The CSI was used to measure CS symptoms as the main mechanism of nociplastic pain (NP) and comprises two parts: part A, with scoring ranges from 0–4, on a continuous scale ranging 0–100, and part B, identifying ten previously diagnosed central sensitization syndromes, on a nominal scale [34,35,36]. Cut-off points are a score ≥40 or severity levels defined as “Subclinical” = 0–29, “Mild” = 30–39, “Moderate” = 40–49, “Severe” = 50–59, and “Extreme” = 60–100 [37,38].

### 2.6. Data Analysis

The sample size for the prognostic model was a priori calculated by G*power 3.1 for linear multiple regression, fixed model, R^2^ increase, using a medium effect size (ρ^2^ = 0.3), a probability of 0.01, and a power of 0.95 for each linear multiple regression analyses with a total number of tested predictors (AASP) of 1 and a total number of predictors of 10 [39]. This resulted in a sample size of 89, and corrected for a loss to follow-up of 20%, 107 participants were needed. When assessing missing data in the analyses, all cases are included if the missing values are completely random, and multiple imputations must be performed if 15% of the predictor data are missing [14,40].

All assumptions for linear and logistic regression analyses were met: the linear relationship between independent and dependent variables; no multicollinearity; the variance in the residuals is constant across the linear model (homoscedasticity of errors); the values of the residuals are normally distributed and independent; no bias due to influential cases. Autocorrelation of the residuals was assessed by the Durbin–Watson test. To assess the overfitting of the data, both in linear regression and univariable and multivariable logistic regression analyses, cross-validation was performed using an 80–20% split of the cases.

Established factors for LBP and chronicity were a priori defined and obtained during data collection and used as adjusting factors in the prognostic model [14]. Using a backward selection approach, factors that seem non-contributing or unnecessary to the model were removed [14,41].

Subsequently, logistic regression analysis using cut-off points of CSI ≥ 40 was performed after assessing a Brier score as a measure for disagreement in a binary outcome and the predictor as a model of fit statistics. Statistics calculated in logistic regression analyses included unstandardized beta-coefficients (B), Hosmer–Lemeshow test (χ^2^), Nagelkerke’s R^2^, Wald statistic, Odds Ratios (ORs) with 95% confidence interval (CI), *p*-values, and Receiver Operator Characteristics (ROC) analyses, and the Area Under the Curve (AUC), representing the overall performance of the model at various threshold settings, by using sensitivity (true positives), and 1-specificity (false positives) analyses. When assessing dichotomous models, ORs were used. Significance was set at α = 0.05. All analyses were performed using IBM Statistics v. 28.0.

## 3. Results

**Figure 2:**
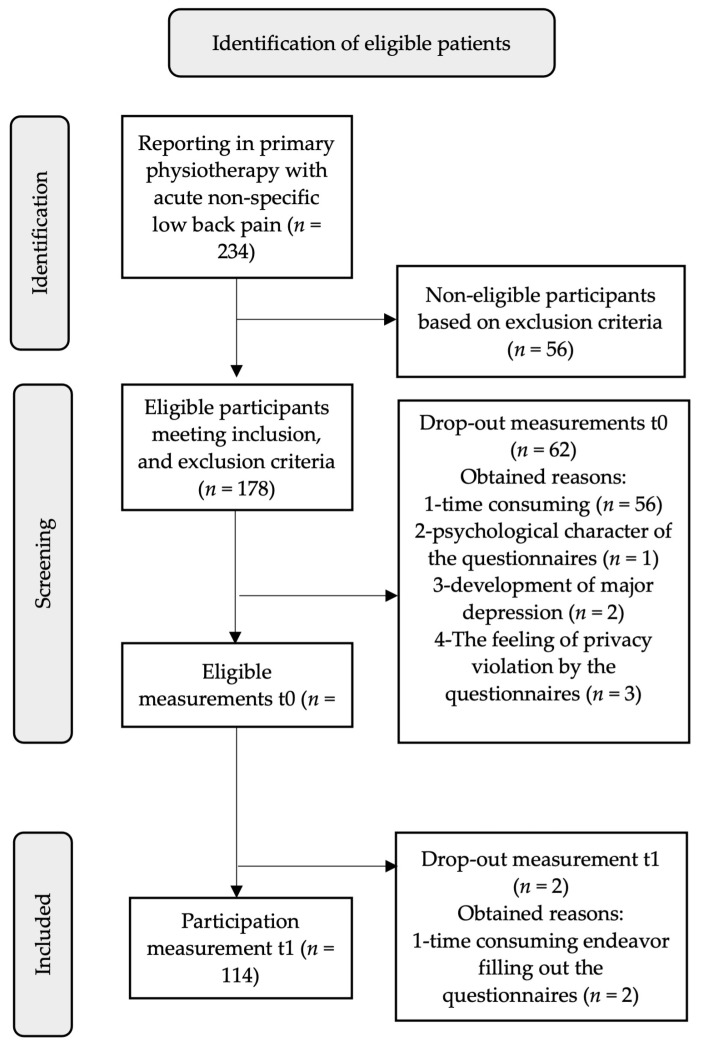
Flow diagram of participation throughout this study.

After recruitment and eligibility assessment, 114 patients completed baseline, and measurement was performed after 12 weeks (*n* = 114) (Figure 2; Table 1; Supplementary Table S1). In <1%, missing data were detected; therefore, they were categorized as completely random and did not influence results [14,40]. All assumptions were met for performing univariable linear regression analyses, multivariable linear regression analyses, and logistic regression analyses. A paired sample *t*-test showed that only the sensory profiles, state, and trait anxiety did not significantly differ over 12 weeks (Table 2). In multivariable linear regression, a Durbin–Watson test was found to be 1.90. To ensure the model is robust, cross-validation is performed (using 5-fold cross-validation, splitting the data into an 80% training set and 20% test set) with a Pearson’s correlation of *r* = 0.62 (*p* ≤ 0.001) for the 80% sample and *r* = 0.56 (*p* = 0.029) for the 20% sample, indicating a robust model and no overfitting of the data in multivariable linear regression. In logistic regression analyses, cross-validation yielded a validation accuracy of *r* = 0.88 and a test accuracy of *r* = 0.81. As suggested by Riley et al. (2019), primary and secondary prognostic factors were assessed in univariable regression analyses to determine their prognostic capability as individual factors [14] (Table 3).

In multivariable regression analyses, analyzing all a priori-defined variables in a single model, only the variables Sensory Sensitive and trait anxiety contributed significantly to the improvement in this model and the prediction of CS symptoms. None of the other factors significantly contribute to the predictive value of this model. Removing the variables in the model by the variable contributing least to the prediction until all variables contributed significantly, known as a backward approach, does not change the interaction between the variables significantly. As a result, multivariable linear regression analyses baseline variable Sensory Sensitive (*p =* 0.00) combined with the baseline variable trait anxiety contributed most to this model. All other prognostic factors did not additionally predict CS symptoms at 12 weeks. In addition, the independent *t*-test is not significant for this model (*p* = 0.25). This model explained 38% (R^2^) of the variance in the prediction of CS symptoms at 12 weeks by Sensory Sensitive, trait anxiety, and a standard error of 4.38 for the model (Table 4). The bootstrap analyses for Sensory Sensitive result in an unstandardized B-value = 0.42 (95%CI = 0.14; 0.71, *p* = 0.01; standard error = 0.15), and for trait anxiety unstandardized B-value = 0.53 (95%CI = 0.31; 0.75; *p* ≤ 0.001; standard error = 0.1), indicating a genuine positive relationship not relying on assumptions of normality or homoscedasticity. The subsequent prognostic model arises from the previous analyses:

CSI_t−1_ = −5.841 + 0.42 (SSv._t0_-score) + 0.53 (STAI.trait._t0_-score)

Coefficient of determination R^2^ = 0.38; standard error = 4.38.
Abbreviations: CSI_t−1_ = Central Sensitization Inventory score at 12 weeks; SSv._t0_-score = Sensory Sensitive score at baseline; STAI.trait._t0_-score = trait anxiety score at baseline.

**Table 4:** Multivariable regression analyses and multivariable binary logistic regression analysis model statistics for Central Sensitization Inventory at 12 weeks.

**Linear Regression Analysis**	**Central Sensitization Inventory**					
	**R^2^**	**F-Ratio**	**B**	**Beta**	***p***	**S.E.**
Model	0.38	31.15 (2; 103)	−5.84		0.19	4.38
SSv			0.42	0.27	0.00	0.13
STAI.trait			0.53	0.45	<0.001	0.10
**Logistic regression analysis**	**Central Sensitization Inventory**					
	**Nagelkerke’s R^2^**	**Wald (df), *p***	**B**		**OR** **(95%CI)**	**S.E.**
Model	0.23	18.79 (1), <0.001	−6.38		0.00 (1.01; 1.14)	1.47
SSv		3.07 (1), 0.04	0.06		1.07 (1.01; 1.14)	0.04
STAI.trait		18.79 (1), <0.001	0.07		1.07 (1.01; 1.14)	0.03

Abbreviations: R^2^ = coefficient of determination; F-ratio = model of fit statistics; B = unstandardized B-value; Beta = Standardized Coefficients Beta; *p* = level of significance; S.E. = standard error; df = degrees of freedom; OR = Odds Ratio; SSv = sensory profile Sensory Sensitive; STAI.trait = trait anxiety.

**Figure 3:**
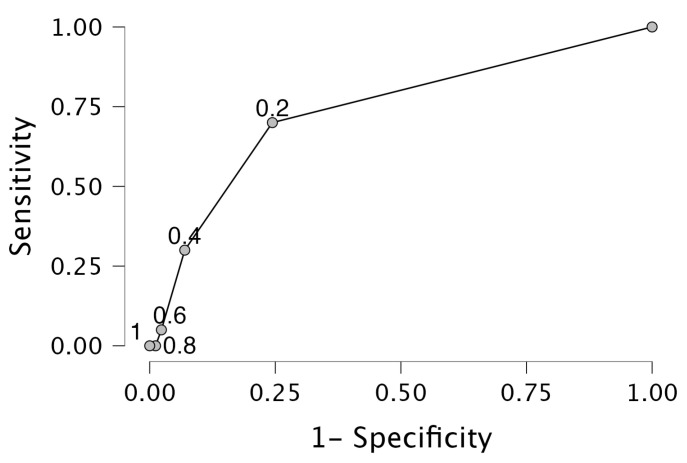
Receiver Operator Characteristic (ROC) analyses. The Receiver Operator Characteristic indicates discrimination of the probability of patients with and without Central Sensitization after 12 weeks by baseline sensory profile Sensory Sensitive and baseline trait anxiety, resulting in AUC = 0.8, sensitivity = 0.10, specificity = 0.98.

In logistic regression, Sensory Sensitive and trait anxiety contribute most to the risk of patients developing CS symptoms at 12 weeks. The Hosmer–Lemeshow test shows that the variables are independent of each other (χ^2^ = 6.36; df = 7; significance = 0.50), and bootstrap analyses for internal validation of the predictive performance show that the model is a good fit to the data (χ^2^ = 9.44, df = 2, *p* = 0.00) (Table 4). A Brier score of 0.13 was found when assessing the accuracy of probabilistic predictions (ranging from 0 = perfect accuracy to 1 = perfect inaccuracy). The model explained 23% (Nagelkerke’s R^2^) of the variance in the persistence of CS symptoms as correctly classified, with significant Wald statistics indicating that the variables are useful in the model, and an OR of 1.07 was adjusted for Sensory Sensitive and trait anxiety. In ROC analyses, the Area Under the Curve (AUC) = 0.8, indicating a good ability of the model to discriminate between CS symptoms and no CS symptoms with sensitivity = 0.10 and specificity = 0.98 (Figure 3).

## 4. Discussion

No previous study showed a prognostic model predicting CS symptoms in the LBP population after 12 weeks. In support of our hypotheses, in patients with ALBP, CS symptoms at 12 weeks can be predicted by Sensory Sensitive and trait anxiety. Hypothesizing that maladaptive behavior, based on sensory profiles, such as avoiding sensory stimulation, may contribute to the development of CS symptoms, only Sensory Sensitive is significant in the multivariable linear model, indicating that the combination of a low sensory threshold and a passive response to sensory overstimulation contributes most in predicting CS symptoms at 12 weeks.

On continuous data, R^2^ = 0.38, indicating 38% of the variance in CS symptoms was accounted for by the predictive factors of Sensory Sensitive and trait anxiety [42]. In most clinical studies, R^2^ ranges from 0.2 to 0.4, indicating that R^2^ = 0.38 is on the higher end and is a substantial fit for the data [42,43]. Thirty-eight percent of the variation was accounted for by Sensory Sensitive and trait anxiety, consequently leaving 62% of the variation not explained by the model. The unexplained variation in this model includes all possible unidentified factors influencing CS symptoms at 12 weeks, containing known factors and factors that are currently not yet identified [43,44]. Identifying all factors and, as a result, explaining all variances is considered a pipe dream but results in an R^2^ = 1.00. Obtaining the contribution in the prediction of CS symptoms, the unstandardized beta-value of trait anxiety (B = 0.53) is larger than the unstandardized beta-value of Sensory Sensitive (B = 0.42). The B-value clinically results in a 0.53-point increase in CSI for every unit increase in trait anxiety (STAI.DY.2), and every point increase on the Sensory Sensitive (AASP) increases the CSI by 0.42 points. Due to that, trait anxiety has a larger contribution to the outcome. When assessing the clinical predictive capability of the model, the outcome ideally covers the clinically relevant range of the CSI, ranging from CSI ≥ 25 to CSI ≥ 60 [37,38,45]. Although a general cut-off point is suggested and used in our study, the current perception is that we are aware of a gradual transition in CSI scores, which may guide clinical reasoning. In addition, recently, different cut-off points have been determined for males and females [45]. Including the minimum values (Sensory Sensitive = 15 points; STAY.DY.2 = 20 points) and maximum values (Sensory Sensitive = 75 points; STAI.DY.2 = 80 points) of the variables in the model, the model’s lowest and highest obtainable CSI values can be obtained. As a result, the clinical predictive capability of the prognostic model based on continuous data ranges from 17 to 74, indicating that clinically meaningful cut-off points or severity levels can be identified by the model [37,38]. All factors are previously established factors for LBP and chronic musculoskeletal pain conditions, but some did not contribute to that prediction in the population used in this study (age, duration of LBP, NPRS, catastrophizing, helplessness, and rumination) [23,44]. An explanation may be that CS symptoms are the predicted factor and not pain severity in LBP, although patients with LBP were the target population, and known factors of pain severity in LBP have also a priori been included [23,44]. Also, the target population was patients with LBP in the acute phase, and therefore, when obtaining the data, CS cannot yet be assumed, and a combination of known CS symptoms and LBP prognostic factors have been included in this study and resulted in non-contributing factors. To identify CS and heightened sensitivity symptoms in musculoskeletal pain, Nijs et al. (2014) suggest an algorithm containing the following factors: disproportional pain experiences; diffuse pain distribution; and a CSI score ≥ 40, although other cut-off points and severity levels are suggested [37,46]. Notably, the CSI is the only quantifiable factor on a continuous scale identifying CS symptoms, and the other factors in the algorithm are measured on a dichotomous scale [46]. Therefore, only the CSI after 12 weeks is considered in predicting CS symptoms in the model.

When assessing the persistence of CSI scores between baseline and at 12 weeks, the number of patients with a CSI score ≥ 40 decreases significantly over 12 weeks (T = 3.28; *p* ≤ 0.001). Half of the patients with a CSI ≥ 40 at baseline persisted in heightened CSI scores, and 4% increased above the cut-off score, suggesting that CSI scores over time are not related to each other in acute pain stages. Peripheral hyperalgesia can produce a heightened score on the CSI questionnaire in the acute phase, which normally restores within a few weeks [5]. Persistence of heightened sensitivity can become maladaptive and can be considered CS [10].

Using baseline CSI scores as an independent prognostic factor to predict CSI scores is not preferable, as heightened sensitivity is already present to affect CSI scores at baseline. Therefore, no genuine effect can be determined after 12 weeks [47]. Concerning patients with ALBP, peripheral sensitization in the acute pain stage is a normal response rather than maladaptive CS and can be considered a confounding factor or an effect modifier, whereas heightened baseline CSI scores can be obtained [5,48,49]. Therefore, pain experiences must be present for at least 3 months to exceed the acute pain stage; otherwise, CS cannot be assumed [3,49].

In line with our findings, as a major underlying mechanism for CS, ALBP somatization and pre-morbid high Sensory Sensitivity predict CS development, whereas sensory profiles with Low Registration and Sensation Sensitive contribute to the prediction of CS symptoms [10,12,44]. However, in our study, a cohort has been followed for over 12 weeks, and the study of Clark et al. (2019) used a cross-sectional observational study design in a CLBP population [10].

### 4.1. Clinical Implications

Interpretation of the CSI questionnaire can be continuous, dichotomous, and categorical [36]. When analyzing the data, it is recommended to use continuous data, rather than dichotomized data [14,50]. Dichotomous analysis has been added because it is common in clinical practice [14,50]. However, when using dichotomized data, a cut-off point must be determined, which is often arbitrarily, and scores below the cut-off point are considered the same as scores well below the cut-off point [14]. Also, dichotomizing the data deteriorates the ability to detect the genuine predictive performance of prognostic models [14]. However, in clinical practice, a dichotomous model may be more helpful in interpreting the outcome of a questionnaire [34,36]. In logistic regression analyses, ROC analyses result in a sensitivity = 0.10 and a specificity = 0.98. A sensitivity of 0.10 indicates that 10% of the people with CS symptoms are correctly identified by the model, leaving 90% of the patients with positive CS symptoms unidentified. Moreover, the specificity of 0.98 indicates that 98% of the people without CS symptoms are identified correctly, leaving 2% of the patients incorrectly diagnosed. Consequences for clinical practice are an almost perfect prediction for patients not developing CS symptoms, but they are poorly predicted for people developing CS symptoms. In clinical practice, this is useful for identifying patients with ALBP who are at risk.

To prevent chronicity, clinical practice guidelines suggest top–down interventions in the acute stage, including cognitive behavioral therapy principles and behavioral interventions to modify (increase or decrease) bottom–up sensory input, minimizing sensory discomfort and stress [1,51,52,53]. To avoid overstimulation pain, neuroscience education may be the management of choice based on sensory preferences, expressed as Sensory Profiles that align with clinical practice guidelines for LBP, which emphasize the importance of cognitive behavioral therapy principles and behavioral interventions [1,19,52]. Implemented in the suggested models, a specific CSI score or an odds ratio identify patients at risk for developing CS symptoms; therefore, maladaptive behavior could be corrected in the acute stage of an LBP episode to prevent chronicity [10].

### 4.2. Limitations

One-third of the invited patients (*n* = 62) declined participation, providing the following reasons: excessive time investment; the psychological nature of the questionnaires; some patients developed major depression; and some patients experienced a violation of their privacy by filling out the questionnaires. This may have led to participation bias, as these specific characteristics may have influenced the results. When inquiring about specific psychological diseases or preferences, some patients did not feel free to answer, as they considered physiotherapy of a musculoskeletal nature and their psychological well-being as very private. Therefore, an extensive explanation of the bio-psychological model was used to prevent participation bias as much as possible, and all eligible patients were invited to participate. It is important that every patient can participate in this study; however, by using PROMs, health literacy is recognized as a major determinant of health disparities and is associated with poorer health outcomes. Although minimally, the results may be biased by that.

In clinical practice, the CSI is recommended to measure CS symptoms, in contrast to quantitative sensory testing, which is mostly used in research settings [44,54,55]. Moreover, quantitative sensory testing equipment is very expensive, testing is time-consuming, and it requires specific training. Therefore, it is difficult to integrate into clinical practice. The CSI is commonly used; the psychometric properties have extensively been researched and generate valid and reliable data [3,34,36,38,46,54,55]. In addition, using the CSI questionnaire is the only quantifiable measure on a continuous scale to use in regression analyses.

Identification of CS goes beyond musculoskeletal pain conditions and includes pain experiences not entirely explained by a clear origin or nociceptive input, widespread pain experiences, and Central Sensitization Inventory (CSI) score ≥ 40 [3,37,38,46,55].

A limitation can be that 71% of the patients reported a previous episode of LBP during their lifetime. Recurrent episode(s) of LBP may indicate underlying chronicity, despite all patients reporting a pain-free period of >12 weeks before the current episode of LBP; therefore, it is diagnosed as ALBP [19].

### 4.3. Recommendations

The next step is conducting a type 4 predictor of treatment effect study to assess a patient’s response to a specific treatment. Researching behavioral interventions in LBP populations based on sensory profile assessment in predicting CSS may lead to a further understanding of LBP development over time.

## 5. Conclusions

This study showed that Sensory Sensitive as one of the Sensory Profiles, combined with anxiety, predict central sensitization symptoms after 12 weeks in patients with ALBP. As central sensitization is the main mechanism behind nociplastic pain, both models may predict chronic pain conditions.

## Figures and Tables

**Table 1:** Clinical characteristics of patients with acute low back pain.

	Baseline
Male (*n*) (%)	64 (56.1)
Age (yrs.) (SD)	45 (11.1)
Widespread pain (*n*) (%)	41 (36.0)
Duration of LBP (wks.) (SD)	3.0 (1.5)
Severity of LBP (NPRS) (SD)	6.1 (1.9)
Severity of leg pain (NPRS) (SD)	1.6 (2.3)
Recurrent episodes (*n*) (%)	81 (71.1)

Abbreviations: yrs. = years; SD = standard deviation; *n* = number of participants; wks. = weeks; LBP = low back pain; NPRS = Numeric Pain Rating Scale.

**Table 2:** Variables at baseline and 12 weeks.

Variable	Baseline	12 Weeks	Paired Sample *t*-Test		
	Mean (SD)	Mean (SD)	Mean Difference	95%CI	Two-Sided (*p)*
CSI-A	30.33 (12.40)	27.33 (12.28)	−3.00	1.09; 4.41	<0.001
*Sensory Profiles*					
Low Registration	27.63 (7.18)	27.09 (7.13)	−0.54	−0.65; 0.95	0.71
Sensation Seeking	45.18 (7.40)	45.19 (8.40)	0.01	−0.80; 0.97	0.85
Sensory Sensitive	31.48 (8.07)	31.29 (8.80)	−0.19	−0.96; 0.85	0.90
Sensation Avoiding	31.81 (8.97)	31.08 (8.36)	−0.73	−0.40; 1.40	0.27
NPRS	6.06 (1.95)	1.96 (1.95)	−4.10	3.91; 4.91	<0.001
STAI state	35.67 (11.30)	34.82 (11.50)	−0.85	−0.72; 2.90	0.24
STAI trait	37.96 (10.37)	36.86 (10.84)	−1.07	−0.65; 2.46	0.25
BDI	8.94 (8.47)	6.78 (6.94)	−2.16	1.35; 4.15	<0.001
PCS cat.	10.25 (9.29)	7.52 (9.29)	−2.73	1.17; 4.45	<0.001
PCS hel.	4.05 (4.29)	2.84 (3.99)	−1.21	0.43; 1.91	0.002
PCS rum.	4.33 (3.84)	3.19 (3.79)	−1.14	0.61; 1.98	<0.001
PCS mag.	1.80 (2.11)	1.30 (2.02)	−0.50	3.91; 4.91	<0.001

Abbreviations: sd = standard deviation; 95% CI = 95% confidence interval; *p* = level of significance; CSI-A = Central Sensitization Inventory Part A; NPRS = Numeric Pain Rating Scale; STAI state = State-Trait Anxiety inventory state; STAI trait = State-Trait Anxiety Inventory trait; BDI = Beck Depression Inventory; PCS cat. = Pain Catastrophizing Scale—catastrophizing; PCS hel. = Pain Catastrophizing Scale—helplessness; PCS rum. = Pain Catastrophizing Scale—rumination; PCS mag. = Pain Catastrophizing Scale—magnification; NPRS = Numeric Pain Rating Scale; CSI = Central Sensitivity Inventory.

**Table 3:** Univariable regression analysis on Central Sensitization Inventory at 12 weeks.

	R^2^	F-Ratio	B	*p*	S.E.
LR	0.11	13.22 (1; 104)	0.60	<0.001	0.17
SSk	0.00	0.01 (1; 104)	−0.01	0.93	0.16
SSv	0.22	28.60 (1; 104)	0.71	<0.001	0.13
SA	0.24	31.90 (1; 104)	0.71	<0.001	0.13
Age	0.00	0.09 (1; 104)	0.03	0.76	0.11
Duration	0.00	0.46 (1; 103)	−0.57	0.50	0.83
NPRS	0.00	0.30 (1; 103)	0.34	0.59	0.62
STAI-state	0.26	35.34 (1; 103)	0.55	<0.001	0.09
STAI-trait	0.32	48.08 (1; 104)	0.67	<0.001	0.10
BDI	0.09	10.17 (1; 99)	0.44	0.00	0.14
PCS-cat	0.01	0.80 (1; 101)	0.12	0.37	0.13
PCS-help	0.00	0.04 (1; 102)	0.06	0.84	0.29
PCS-rum	0.00	0.35 (1; 102)	0.18	0.56	0.31
PCS-mag	0.06	6.66 (1; 101)	1.45	<0.001	0.56

Abbreviations: CSI = Central Sensitization Inventory; LR = sensory profile Low Registration; SSk = sensory profile Sensory Sensitive; SSv = sensory profile Sensation Sensitive; SA = sensory profile Sensation Avoiding; Age = age; Duration = duration of low back pain; NPRS = numeric pain rating scale; STAI-trait = trait anxiety inventory; STAI-state = state anxiety inventory; BDI = Beck Depression Inventory; PCS = Pain Catastrophizing Scale—catastrophizing; PCS-help. = Pain Catastrophizing Scale—helplessness; PCS-rum. = Pain Catastrophizing Scale—rumination; PCS-mag. = Pain Catastrophizing Scale—magnifying; R = measure of quality of the prediction; R^2^ = explained proportion of variance; F-ratio (regr; resid) = overall fit of the model; *p* = significance; B = estimated model coefficient; t = statistical significance of the independent variables, S.E. = standard error.

## Data Availability

Data are unavailable due to privacy restrictions.

## Supplementary Materials

The following supporting information can be downloaded at https://www.mdpi.com/article/10.3390/jcm13164677/s1, Table S1: Clinical characteristics at baseline of 114 patients.
