# Supplementary material for: Sensory Profiles Predict Symptoms of Central Sensitization in Low Back Pain: A Predictive Model Research Study
Source: J Clin Med. 2024 Aug 9;13(16):4677. doi: 10.3390/jcm13164677 (PMC11355633; doi:10.3390/jcm13164677)
Supplement: Supplementary file 1 [file jcm-13-04677-s001.zip › jcm-3116439-supplementary.pdf]

SUPPLEMENTARY MATERIALS

Supplement S1. Clinical characteristics at baseline of 114 patients

	Baseline
Male (N) (%)	64 (56.1)
Age (yrs.) (SD)	49 (41.0)
Height (m) (SD)	1.78 (0.09)
Weight (kg) (SD)	84 (15.6)
BMI (kg/m^2) (SD)	26.3 (4.0)
Widespread pain (N) (%)	41 (0.37)
Duration of LBP in weeks (wks.) (SD)	3.00 (1.48)
First episode of LBP (N) (%)	32 (28.1)
Age first episode of LBP (yrs.) (SD)	30 (13.1)
Severity of LBP (NPRS) (SD)	6.06 (1.95)
Severity of leg pain (NPRS) (SD)	1.58 (2.31)
Pain medication	
Yes (N) (%)	69 (61.0)
No (N) (%)	44 (38.6)
Sometimes (N) (%)	31 (27.2)
Anti-inflammatory medication	
Yes (N) (%)	14 (12.3)
No (N) (%)	91 (79.8)
Sometimes (N) (%)	8 (7.0)
Married/ living together (N) (%)	78 (68.4)
Social support (N) (%)	109 (95.6)

Sleep behavior	
I have no trouble sleeping (%)	25 (21.9)
My sleep is slightly disturbed (less than 1 hrs. sleepless) (%)	27 (23.9)
My sleep is mildly disturbed (1-2 hrs. sleepless) (%)	41 (36.0)
My sleep is moderately disturbed (2-3 hrs. sleepless) (%)	18 (15.8)
My sleep is greatly disturbed (3-5 hrs. sleepless) (%)	2 (1.8)
My sleep is completely disturbed (5- hrs. sleepless) (%)	0 (0.0)
Level of education	
High (N) (%)	41 (36.0)
Middle (N) (%)	65 (57.0)
Low (N) (%)	8 (7.0)
Employment (N) (%)	100 (87.7%)
Work environment	
Sedentary (N) (%)	55 (48.2)
Standing (N) (%)	9 (7.9)
Standing work+ lifting (N) (%)	34 (29.8)
Physical heavy (N) (%)	14 (12.3)
Smoking (N) (%)	32 (28.1)
Recurrent episodes (N) (%)	81 (71.1)
Comorbidities (N) (%)	41 (36.0)
Expectation (N) (%)	90 (78.9)

Abbreviations: SD= standard deviation, LBP= low back pain, BMI= Body Mass Index, NPRS= Numeric Pain Rating Scale, PDI= Pain Disability
